# Digital Behavioral Infrastructure for Glucagon-Like Peptide-1 Pharmacotherapy: Viewpoint on Persistence, Tolerability, and Postcessation Durability

**DOI:** 10.2196/104227

**Published:** 2026-09-08

**Authors:** Geoff Cook

**Affiliations:** 1 Friedman School of Nutrition Science and Policy, Tufts University Boston, MA United States

**Keywords:** glucagon-like peptide-1 receptor agonists, GLP-1 receptor agonists, glucagon-like peptide-1 companion, GLP-1 companion, obesity, antiobesity medications, digital health, digital behavior change interventions, medication adherence, weight regain, habit formation, behavioral economics

## Abstract

Glucagon-like peptide-1 receptor agonists (GLP-1 RAs) induce clinically meaningful weight loss, but their real-world impact is constrained by poor medication persistence, treatment-limiting gastrointestinal adverse effects, and rapid weight regain after cessation. These limitations may be structural rather than incidental: GLP-1 RAs powerfully address appetite biology but do not build the behavioral skills, environmental supports, and routines needed to sustain outcomes when biological pressures revert to baseline. This viewpoint argues that theory-based digital health companion programs are best understood as structural complements to glucagon-like peptide-1 (GLP-1) therapy rather than optional adjuncts, and it articulates a testable mechanistic hypothesis: a pharmacologically enabled “habit window.” We map theoretical determinants from the social cognitive theory (SCT) and behavioral economics (BE) to classes of digital intervention across 3 problems (medication persistence, tolerability, and postcessation durability) and grade the supporting evidence as established, observational, or hypothesized. For each problem, we link candidate SCT- and BE-informed mechanisms (such as self-efficacy and enactive mastery, present bias, defaults, and loss aversion) to specific digital intervention classes and to the studies needed to test them. Because reduced appetitive drive may free cognitive resources and lower the need for food-related self-control, GLP-1 therapy may open a privileged window in which habit formation is easier. Supporting evidence is drawn from adjacent behavioral trials, combined pharmacological and lifestyle trials, and observational engagement data; the observational data are hypothesis generating and subject to selection effects. Digital behavioral infrastructure may help translate the biological effects of GLP-1 therapy into durable behavioral and environmental change, but the “habit window” remains a hypothesis requiring prospective and randomized testing. We outline a research agenda prioritizing randomized trials with postcessation follow-up and mechanistic mediation studies.

## Introduction

Glucagon-like peptide-1 receptor agonists (GLP-1 RAs) have transformed obesity care. In clinical trials, semaglutide 2.4 mg induces mean weight loss of 14.9% at 68 weeks [1] and tirzepatide 15 mg induces reductions of 20.9% [2], with efficacy approaching that of bariatric surgery. However, 3 challenges constrain real-world impact.

First, medication persistence is poor: a longitudinal analysis of 5780 commercially insured adults without diabetes found only 8% remained on glucagon-like peptide-1 (GLP-1) therapy at 3 years [3]. Second, gastrointestinal adverse events drive early discontinuation; 20% to 50% of patients stop within the first year [1,4]. Third, weight regain after cessation is rapid: a 2026 systematic review and meta-analysis in the *BMJ* estimated that patients discontinuing semaglutide or tirzepatide return to baseline weight within approximately 18 months, a rate of weight regain 4 times faster than those discontinuing a behavior program [5].

These limitations share a single structural root. GLP-1 RAs reduce both physiological appetite and the cognitive phenomenon patients describe as “food noise,” but they do not build the behavioral skills, environmental supports, or routines necessary to sustain outcomes when those biological pressures revert to baseline in the absence of the medication. Treated as a stand-alone therapy, GLP-1 RAs may be clinically powerful but behaviorally incomplete when delivered without supports that help patients build durable routines, manage treatment burden, and sustain change over time.

Digital health companion programs deliver behavioral coaching, self-monitoring, personalized feedback, and choice-architectural support alongside pharmacotherapy. We argue that such programs, when theory based, have the potential to combine with the medication for a more durable, systems-based approach. Such programs may be better conceptualized not as optional adjuncts but as structural complements to GLP-1 therapy. A growing number of consumer health companies now offer GLP-1 companion experiences of this type: WeightWatchers has launched a GLP-1 program that pairs medication with structured behavioral coaching [6], and Oura has begun linking GLP-1 support to continuous physiological data through a partnership with a prescribing service [7].

We ground this argument in 2 complementary frameworks: social cognitive theory (SCT), which models reflective behavior change through reciprocal interactions among person, behavior, and environment [8]; and behavioral economics (BE), which addresses the automatic dimensions of decision-making, including present bias, status quo bias, and cue-dependent habits [9,10]. Reviews of digital health interventions show that techniques aligned with these frameworks, including self-monitoring, goal setting, social support, feedback, and prompts, improve outcomes in obesity [11]. Together, the SCT helps explain deliberate and social routes to adherence, while BE helps design choice environments that may make desired behaviors easier, more immediate, and more likely to become automatic.

We draw on 5 constructs in particular, each of which maps to a specific class of digital intervention (Table 1). From the SCT, self-efficacy, defined as a person’s confidence in performing a specific behavior, is the strongest proximal predictor of behavior change and is built most effectively through enactive mastery (small, repeated successes) and vicarious experience (observing similar others succeed) [12]. Reciprocal determinism, the SCT’s premise that personal factors, behavior, and environment continuously shape one another, implies that a well-designed digital environment can make target behaviors more likely and, in turn, more self-reinforcing [13]. From BE, present bias, the tendency to overweight immediate costs relative to delayed benefits, explains why the early and uncomfortable phase of therapy is vulnerable to discontinuation and motivates immediate reinforcement [14]. Status quo bias, the tendency to remain with a default option, can be harnessed by making the supportive path the default [15]. Loss aversion, the disproportionate weight placed on losses relative to equivalent gains, motivates loss-framed feedback, such as streak tracking [16]. We apply these constructs to each of the 3 problems in the sections that follow.

**Table 1:** Evidence base for proposed digital behavioral mechanisms in glucagon-like peptide-1 (GLP-1) pharmacotherapy^a^.

Target problem	Proposed mechanism	Example digital intervention	Evidence status	Needed study
Medication persistence	Present bias, self-efficacy, and social accountability	Injection reminders, adverse effect coaching, peer support, and immediate reinforcement for medication logging	Plausible and supported by behavioral theory and observational engagement data	Randomized controlled trial measuring medication possession ratio, persistence, discontinuation timing, and patient-reported treatment burden
Adverse effects and tolerability	Titration support, dietary adjustment, symptom self-monitoring, and timely clinical escalation	Symptom tracking; dose-escalation education; clinician alerts; and dietary guidance for nausea, vomiting, constipation, and diarrhea	Clinically plausible and limited direct evidence for digital companion programs	Pragmatic trial during dose escalation measuring adverse-event burden, dose-escalation success, discontinuation due to adverse effects, and patient satisfaction
Lean mass preservation	Self-regulation, feedback, loss aversion, and exercise habit formation	Resistance training modules, protein intake prompts, body composition tracking, and streaks or commitment devices	Supported indirectly by exercise, body composition, and behavioral economics literature and limited GLP-1^b^–specific digital evidence	Trial measuring fat mass, fat-free mass, strength, physical function, adherence to resistance training, and nutrition behaviors during GLP-1 therapy
Postcessation durability	Habit automaticity, identity-based motivation, relapse prevention, and environmental restructuring	Cue-routine-reward planning, self-monitoring, relapse planning, identity-based goal setting, and transition support before discontinuation	Supported indirectly by S-LiTE^c^, habit formation literature, and observational data from companion programs	Longitudinal trial with postcessation follow-up measuring weight maintenance, habit automaticity, food noise, self-efficacy, cardiometabolic outcomes, and quality of life

^a^Evidence categories reflect the author’s interpretation of the current literature and are intended to guide future research rather than imply established causal efficacy of digital companion programs.

^b^GLP-1: glucagon-like peptide-1.

^c^S-LiTE: Subcutaneous Liraglutide and Treadmill Exercise.

**Figure 1:**
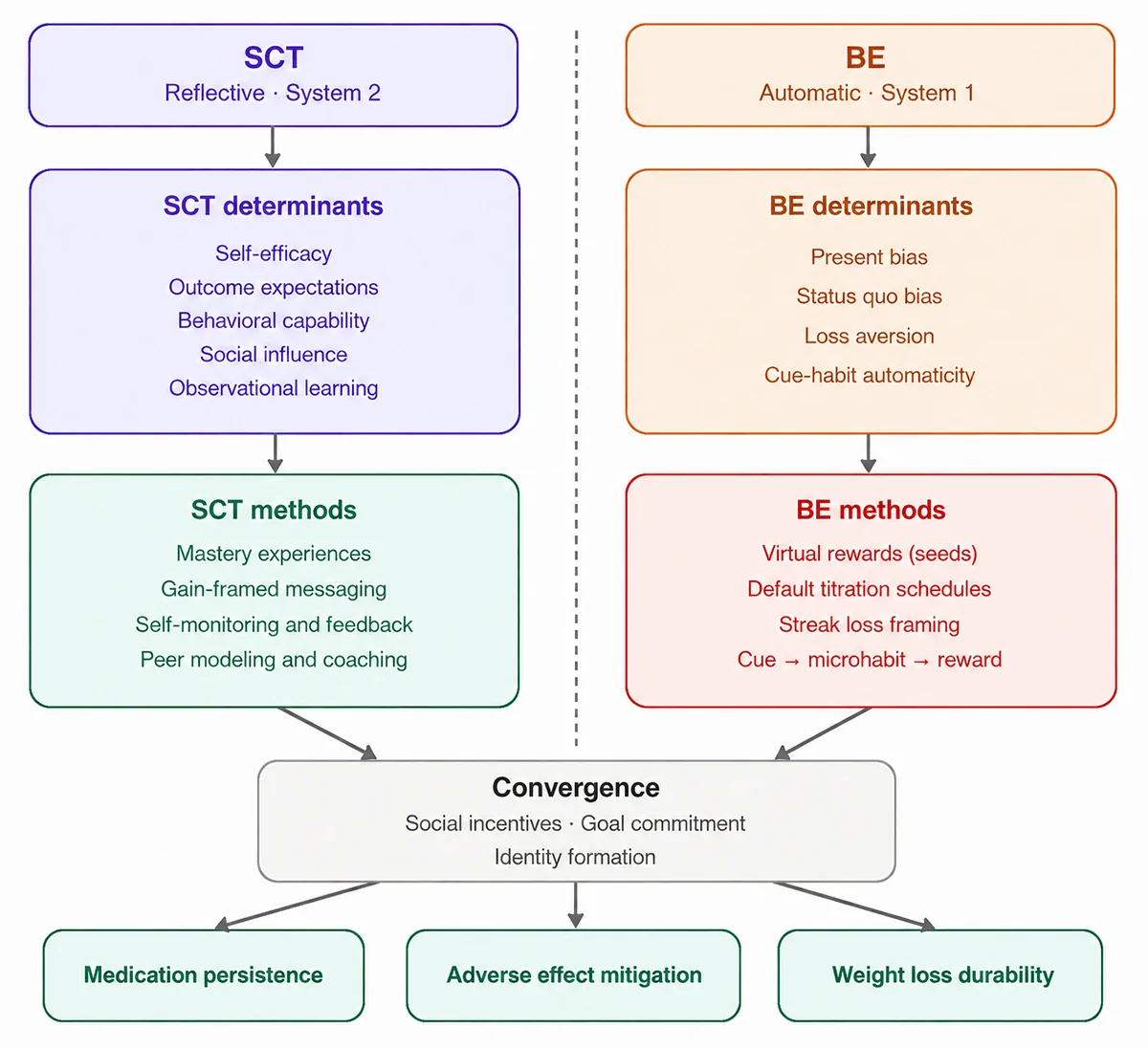
Integrative framework mapping the social cognitive theory (SCT) and behavioral economics (BE) constructs to classes of digital interventions and target outcomes. Both reflective (SCT or system 2) and automatic (BE or system 1) pathways are shown, converging on 3 target outcomes: medication persistence, adverse effect mitigation, and postcessation weight loss durability. In the figure, the virtual reward points referenced under the BE methods are depicted as “seeds.”.

In what follows, we consider medication persistence, adverse effect management, and postcessation durability in turn, mapping theoretical determinants to potential digital interventions (Figure 1). We use the term “privileged window” as a mechanistic hypothesis and not as an established clinical phenomenon; prospective studies are needed to test whether reductions in food noise mediate improvements in self-efficacy, habit automaticity, and postcessation durability. Throughout, we distinguish among evidence that is established, observational, or hypothesized.

## Improving Medication Persistence

Patients immediately experience the costs of GLP-1 therapy, which include injection-site discomfort, nausea, and financial burden, while benefits accrue slowly. From a BE perspective, this mismatch reflects present bias: immediate costs are weighted more heavily than delayed benefits. The visceral influence model by Loewenstein [17] describes how “hot state” somatic experiences further narrow attention to momentary discomfort. Decisions to discontinue may therefore be shaped by momentary discomfort as well as by considered evaluations of long-term benefit.

A complementary SCT-framed account highlights self-efficacy, defined as confidence in executing a specific behavior, as the strongest proximal predictor of both initiation and maintenance [12]. GLP-1 persistence often requires managing injections, navigating insurance, adjusting doses, and sustaining motivation through plateaus; low self-efficacy for these tasks is plausibly associated with discontinuation.

GLP-1 RAs may themselves enhance self-efficacy through a neurobiological pathway. By quieting food noise, they can reduce the cognitive demands of resisting food cues: the INFORM survey of 550 semaglutide users found that constant food-related thoughts decreased from 62% before treatment to 16% after treatment initiation [18]. This reduction in appetitive rumination may free cognitive resources and make behavioral change feel more achievable, consistent with evidence that food cravings consume limited cognitive resources and interfere with competing cognitive demands [19].

We hypothesize that digital companion programs can address both barriers simultaneously. One potential SCT-informed implementation would promote self-efficacy for injection by facilitating enactive mastery through step-by-step injection tutorials and helpful adverse effect management advice. Meanwhile, a potential BE-informed implementation would award a digital reward immediately upon logging each injection, making benefits tangible at the moment of behavior.

## Managing Tolerability and Preserving Health During Weight Loss

### Supporting Dose Escalation and Tolerability

Gastrointestinal adverse events, including nausea, vomiting, and diarrhea, are among the primary drivers of early discontinuation, particularly during dose escalation [4]. This renders the early titration period a critical window for digital support.

Companion programs may mitigate adverse effects through 2 complementary mechanisms. First, an SCT-informed intervention could enable vicarious mastery experience through a peer forum where users observe others persisting through early adverse effects and sharing their strategies for success. The distinct value of an in-app forum over the large, freely available GLP-1 communities on Reddit, Facebook, and Discord would lie in clinical moderation, integration with users’ self-monitoring data, and active misinformation control. Without these functions, existing public communities may already meet this need, and companion apps should focus on complementary roles. Second, a BE-informed intervention could leverage status quo bias by setting a slower titration schedule as the default (eg, holding each dose an additional 4 to 6 weeks before escalation) so that the default pathway favors tolerability while preserving clinician oversight and patient choice. Any such default would be expected to remain within the manufacturers’ approved dose-escalation schedules for semaglutide and tirzepatide; a schedule that departed from labeling would constitute off-label prescribing and would be based on clinical judgment. A companion platform should therefore present a slower titration only as a clinician-authorized option rather than an automatic default, leaving prescribing authority with the treating clinician. Indirect support for this combined approach comes from the STEP 3 trial, in which semaglutide plus intensive behavioral therapy achieved 16.0% weight loss with gastrointestinal-related discontinuation of only 3.4%, compared with 4.5% in STEP 1 that had less behavioral support [20]. However, because comparisons with STEP 1 are cross-trial and nonrandomized, these data should be treated as suggestive rather than causal. Two further cautions apply. The behavioral arm of STEP 3 comprised up to 30 in-person sessions with a registered dietitian, a more intensive and different modality than a digital companion, so the comparison does not speak specifically to digital scaffolding, and the absolute difference in gastrointestinal-related discontinuation (3.4% vs 4.5%) is small and was reported without a CI or significance test, further limiting its interpretive value.

### Preserving Lean Mass During Weight Loss

Another concern is muscle loss. GLP-1 RAs may preferentially reduce fat mass, but approximately 25% to 40% of total weight lost can come from fat-free mass [21], raising clinical concern for sarcopenic obesity [22]. Digital SCT-informed interventions may support lean mass preservation by operationalizing cognitive self-regulation processes of the SCT, enabling self-monitoring of lean mass loss via smartphone-based 3D body composition imaging [23] while providing resistance training through in-app videos accessible to people with obesity [24]. Beyond body weight and body composition, companion programs can support longitudinal monitoring of obesity-related comorbidities during and after treatment by integrating home blood pressure readings, connected glucometer or laboratory hemoglobin A_1c_ data, and lipid or cardiovascular risk measures, thereby surfacing clinically meaningful changes to both patients and clinicians rather than focusing on weight alone.

Meanwhile, through a BE-informed lens, the same resistance training videos could be framed in the form of loss aversion operationalized through streak tracking, in which the anticipated loss of a streak (ie, a count of consecutive weeks of adherence to resistance training) is intended to motivate continued participation. Patel et al [25] found that loss-framed incentives significantly increased the number of days participants met a step goal compared with gain-framed incentives.

## Protecting Weight Loss After Cessation

For most patients, GLP-1 therapy is not indefinitely sustained, and the weight loss it induces is quickly regained after medication cessation. In the STEP 1 extension, participants regained two-thirds of their prior weight loss within 1 year of semaglutide withdrawal [26]. Meanwhile, the *BMJ* systematic review and meta-analysis reported that patients who lost weight through behavioral programs regained it 4 times more slowly than those discontinuing medication [5].

GLP-1 companion behavior programs may mitigate this weight regain by taking advantage of the reduction in food noise associated with GLP-1 RA pharmacotherapy. Indirect empirical support comes from the S-LiTE (Subcutaneous Liraglutide and Treadmill Exercise) trial. After an 8-week low-calorie diet, 195 adults were randomized to 1 year of maintenance with exercise, liraglutide, combined treatment, or placebo [27]. The combination group achieved the greatest additional weight loss (−9.5 kg vs placebo; *P*<.001) [27]. At the 1-year posttreatment follow-up, participants who received combination treatment experienced 6.0 kg less weight regain than those in the liraglutide-only arm, and a greater proportion maintained ≥10% weight loss 1 year after treatment cessation [28]. These findings are consistent with the hypothesis that habits built during pharmacotherapy persist after medication cessation, whereas pharmacological treatment alone may leave some patients without sufficient behavioral infrastructure to resist regain.

An SCT- and BE-informed companion behavioral intervention may leverage reductions in food noise and food-related self-control demands to build new habits and routines. For example, a tool focused on creating routines, where each routine contains a cue, a behavior, and a reward, would enable choice-architecture design during a privileged pharmacological period. Through the SCT lens, these routines would operationalize goal setting and behavior planning, such that each completion supplies a low-risk enactive mastery experience that strengthens self-efficacy. From a BE perspective, attention to cues and immediately reinforcing rewards may support repetition until the behavior becomes more automatic. Lally et al [29] demonstrated that new behaviors reach 95% of peak automaticity after a median of 66 days of consistent performance. That estimate is a median for relatively simple daily behaviors, with individual times to automaticity ranging widely (approximately 18-254 days), so the figure is illustrative rather than a fixed target for the more complex routines relevant here.

A 6- to 12-month treatment period may provide sufficient time for multiple habits to progress toward automaticity, provided that repetition is consistently scaffolded. Identity congruence also matters: health behaviors are adopted and sustained more durably when perceived as identity-consistent [30]. The well-established “fresh start effect,” in which temporal landmarks that signal a new beginning increase aspirational goal pursuit, offers a basis for expecting such a shift, and the initiation of GLP-1 therapy can be conceptualized as a candidate temporal landmark to be tested in this role [31,32]. These observations are consistent with patient-reported improvements in physical functioning and psychosocial well-being from the STEP trials [33].

The mechanisms described above vary in evidentiary maturity. Some are supported by randomized trial evidence from adjacent domains, others by observational engagement data, and others remain mechanistic hypotheses. Table 1 summarizes the proposed pathways, candidate digital interventions, current evidence status, and studies needed to test each mechanism.

## Illustrative Internal Analyses

The following analyses come from one commercially available companion platform (Noom). They are internal, observational, company generated; have not undergone peer review; and are highly susceptible to healthy-user and selection (“motivated-user”) bias: members who engage more with an app likely differ systematically in motivation, resources, and baseline adherence, independent of any effect of the program. We therefore present them only as directional illustrations of the framework above, not as evidence of efficacy, and the central argument of this viewpoint does not depend on them.

In a January 2026 analysis of 30,239 members, those in the highest app engagement quartile remained on medication about 2.2 times longer than those in the lowest quartile (6.2 vs 2.8 months), and members with at least 1 peer connection were retained approximately twice as long as those without a peer connection [34]. A persistence of 6.2 months is itself modest in absolute terms, underscoring that these figures describe an association rather than a demonstration of durable persistence. In a separate report, members who maintained high engagement during therapy retained more than 80% of their weight loss at 6 months after discontinuation, and 81% described a sense of a “fresh start,” while 94% reported becoming “more mindful eaters” [35].

Distinguishing any genuine program effect from motivated-user selection will require prospective designs: randomization to companion support vs usual care, active-comparator apps, and analyses that adjust for baseline motivation and engagement propensity. Until such studies exist, these internal figures should be read as hypothesis generating only.

## Synthesis: An Integrated Model

Taken together, these mechanisms operationalize SCT’s reciprocal determinism, the feedback loop among person, behavior, and environment. Self-efficacy drives adherence, adherence strengthens confidence and outcome expectations, and social and choice-architectural features reduce barriers while enriching the environment in which the next behavior occurs. Effortful adherence can, over time, progress toward a self-reinforcing habit system that persists after pharmacotherapy is withdrawn.

## Counterarguments and Risks

Digital behavioral infrastructure should not be interpreted as a substitute for long-term pharmacotherapy. For many patients, GLP-1 RAs may appropriately function as long-term treatment for a chronic disease, with behavioral support serving to improve persistence, tolerability, quality of life, and cardiometabolic health rather than to enable discontinuation.

Digital programs also carry risks if poorly designed. Excessive tracking, frequent prompts, or complex engagement requirements may increase cognitive burden, particularly for patients already managing medication access, adverse effects, stigma, cost, and comorbidities. Observational associations between app engagement and improved outcomes may also reflect motivated-user bias rather than causal effects; more engaged users may differ systematically from less engaged users in motivation, resources, or baseline adherence.

Companion programs should therefore be evaluated as supportive interventions that enhance treatment outcomes while avoiding any implication of patient blame. Weight regain, discontinuation, and adherence challenges reflect interactions among biology, environment, social context, medication tolerability, cost, and behavior. The ethical goal of digital support is to scaffold behavior change, not shift responsibility for treatment outcomes onto patients.

A further risk is that the digital tools themselves become a source of burden. App-based programs commonly show rapid attrition, and frequent push notifications, streak mechanics, and daily logging can add cognitive load for patients already managing adverse effects, cost, and stigma. These demands are not evenly distributed, as they presuppose digital literacy and reliable device and data access; poorly designed programs therefore risk widening rather than narrowing disparities. Companion tools should default to the lightest effective touch; keep tracking optional; and be evaluated explicitly for differential engagement and outcomes across socioeconomic, age, and disability groups.

## Evidence Gaps and Research Agenda

Although the theoretical rationale for digital behavioral infrastructure is strong, the evidence base remains incomplete. Existing data are drawn largely from adjacent behavioral intervention literature, clinical trials of combined pharmacological and lifestyle treatment, and observational engagement analyses. These sources support plausibility but do not establish whether digital companion programs causally improve GLP-1 persistence, tolerability, or postcessation durability.

Direct randomized evidence specific to app-based GLP-1 companion programs does not yet exist. The closest trial evidence comes from digital behavior change programs without a pharmacological component: in a randomized controlled trial of 427 adults with overweight or obesity, participants in a digital weight management program continued to lose weight over the 52 weeks following a 16-week intervention, whereas an education-only control group did not [36]. Real-world cohorts pairing app-based services with GLP-1 or GLP-1–gastric inhibitory polypeptide therapy report larger losses among more engaged users but share the same confounding biases previously discussed [37]. Establishing causal benefit will require trials that randomize the companion program itself.

Future studies should test GLP-1 pharmacotherapy with and without theory-informed digital companion support in randomized trial designs. Prespecified outcomes should include medication persistence, dose-escalation success, adverse-event burden, weight maintenance, body composition, cardiometabolic risk markers, patient-reported quality of life, and treatment burden. Trials should also include follow-up after medication discontinuation, as the durability question cannot be answered during active pharmacotherapy alone.

Mechanistic studies are also needed. If GLP-1 therapy creates a “habit window,” then reductions in food noise should prospectively mediate improvements in self-efficacy, habit automaticity, identity-based motivation, and weight maintenance. These pathways should be measured directly rather than inferred from app engagement alone.

Finally, future research should examine heterogeneity of benefit. Digital programs may increase burden for some patients if they require excessive tracking, frequent engagement, or high digital literacy. Studies should therefore evaluate equity, accessibility, engagement heterogeneity, and differential outcomes across socioeconomic, racial, ethnic, linguistic, age, and disability groups. Companion programs should be assessed not only for whether they improve average outcomes but also for whom they work, under what conditions, and at what cost to the patient experience. Because companion programs such as Noom and its peers are largely paid consumer products that run on commercial data, 3 questions deserve further attention: cost-effectiveness and affordability relative to the pharmacotherapy they accompany; the privacy and secondary-use implications of the sensitive health data these platforms collect; and equitable access across income, insurance, and digital literacy strata, so that a structural complement does not become available only to those already advantaged.

## Conclusions

GLP-1 RAs represent a major advance in obesity treatment, but their long-term population impact will depend on more than pharmacological efficacy. Patients must be able to persist with treatment, manage tolerability challenges, preserve functional health during weight loss, and sustain behavior change when treatment is interrupted or discontinued. These challenges reflect the limits of pharmacotherapy alone in a chronic biopsychosocial disease shaped by biology, behavior, environment, and social context.

Digital behavioral infrastructure may offer a scalable complement to GLP-1 therapy. Guided by the SCT and BE, companion interventions can support reflective processes such as self-efficacy, goal setting, and social accountability, while also shaping automatic processes through cues, defaults, immediate reinforcement, and habit formation. In this model, digital support does not replace pharmacotherapy; it may help translate the biological effects of pharmacotherapy into more durable behavioral and environmental change.

The concept of a pharmacologically enabled “habit window” remains a hypothesis, not an established clinical fact. GLP-1s may reduce the biological friction of behavior change by quieting appetite and food-related rumination. The next task for digital health is to determine whether that opening can be converted into durable routines, healthier environments, and lasting cardiometabolic benefit.

## Abbreviations

BE: behavioral economics
GLP-1: glucagon-like peptide-1
GLP-1 RA: glucagon-like peptide-1 receptor agonist
SCT: social cognitive theory
S-LiTE: Subcutaneous Liraglutide and Treadmill Exercise

## Data Availability

The Noom data referenced [34,35] are derived from internal company analyses summarized in company reports; these analyses are observational and have not been independently peer reviewed. Deidentified aggregate data supporting these summaries are available from the corresponding author upon reasonable request, subject to company approval.
